# Emergent dynamics of extremes in a population driven by common information sources and new social media algorithms

**DOI:** 10.1038/s41598-019-48412-w

**Published:** 2019-08-15

**Authors:** N. F. Johnson, P. Manrique, M. Zheng, Z. Cao, J. Botero, S. Huang, N. Aden, C. Song, J. Leady, N. Velasquez, E. M. Restrepo

**Affiliations:** 1 Physics Department, George Washington University, Washington D.C., 20052 USA; 2 Physics Department, University of Miami, Miami, FL 33126 USA; 3 Mendoza College of Business, University of Notre Dame, Notre Dame, IN 46556 USA; 4 Elliott School of International Affairs, George Washington University, Washington D.C., 20052 USA

**Keywords:** Statistical physics, Computational science

## Abstract

We quantify how and when extreme subpopulations emerge in a model society despite everyone having the same information and available resources – and show that counterintuitively these extremes will likely be enhanced over time by new social media algorithms designed to reduce division. We verify our analysis mathematically, and show it reproduces (a) the time-dependent behavior observed in controlled experiments on humans, (b) the findings of a recent study of online behavior by Facebook concerning the impact of ‘soft’ and ‘hard’ news, (c) the observed temporal emergence of extremes in U.S. House of Representatives voting, and (d) the real-time emergence of a division in national opinion during the ongoing peace process in Colombia. We uncover a novel societal tipping point which is a ‘ghost’ of a nearby saddle-node bifurcation from dynamical systems theory, and which provides a novel policy opportunity for preventing extremes from emerging.

## Introduction

A fascinating debate has opened up about the impact on a society of information and news, both true and false; its sudden global availability as a result of the Internet; and its deepening penetration via online social media (e.g. Facebook)^1–9^. King *et al*.^7,8^ elucidated the power of general media information in terms of activating people to express themselves, while Boxell *et al*’s^1^ empirical study suggests that current divisions are not specifically due to the Internet or social media. Meanwhile Stanley and co-workers had earlier found empirical evidence for ‘crowd’ formation in human populations responding to information broadcast during an economic competition^10^. The impacts of division within a society are widespread, affecting the stories and narratives that individuals choose to share^3,4^, their political affiliations and how they vote^5,11^, and even how society views scientific findings, e.g. Darwinian evolution versus intelligent design, and climate change^12^. On issues for which there is no counter-evidence, a surprisingly large number of people may still take an ‘anti-crowd’ viewpoint, e.g. the many people who believe the world is flat and attended the Flat Earth International Conference^13^. Even within the community of professional scientists, there is a non-zero ‘anti-crowd’ that are skeptical about global warming^14^.

**Figure 1:**
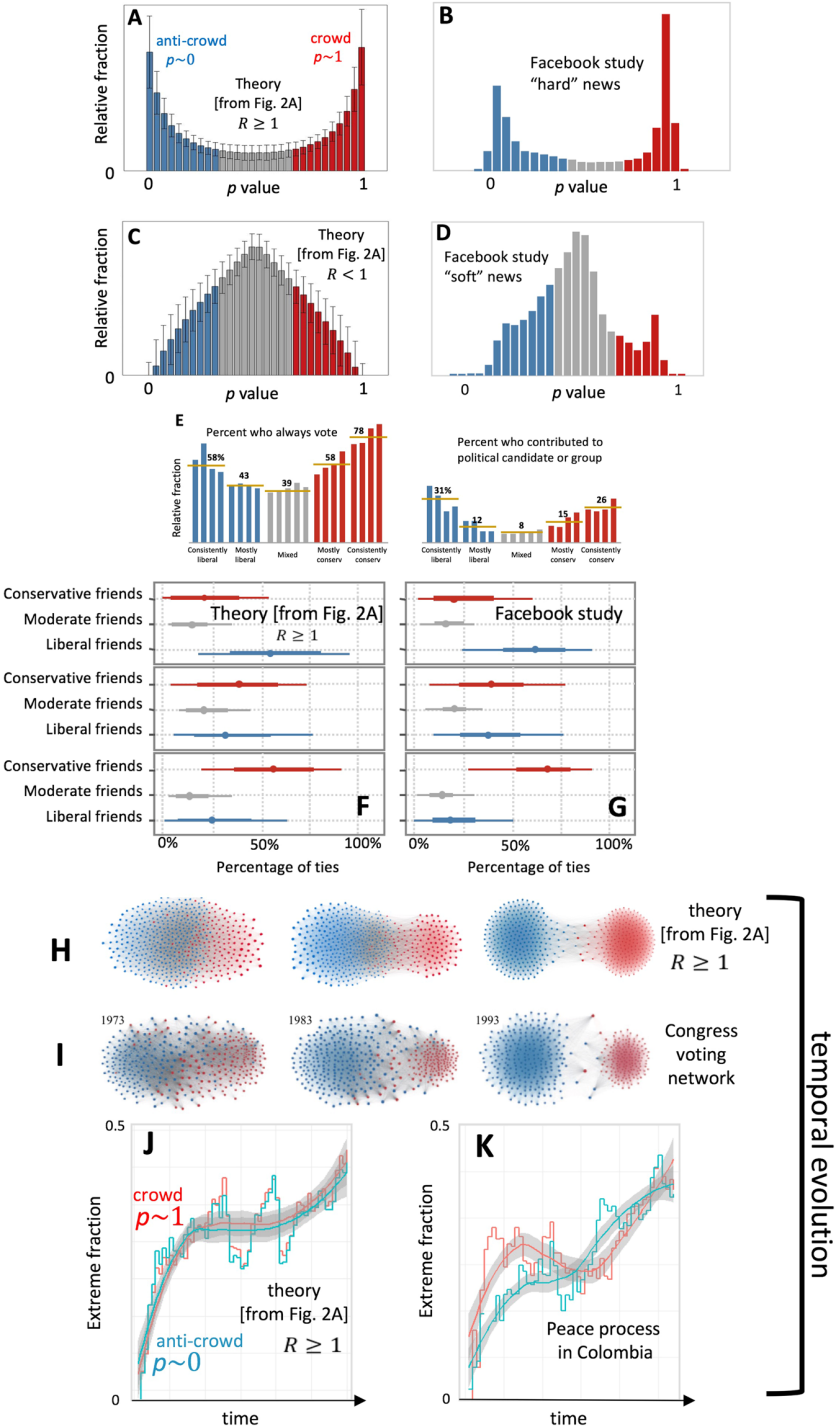
Real-world dynamics of extremes vs. our theory. (**A**–**D**) Heterogeneity distribution *P*(*p*, *t*) showing the relative fraction of population having a particular *p* value (i.e. re-scaled alignment using the terminology of ref.^3^). (**A**,**C**) Our theory for *R* ≥ 1 and *R* < 1 respectively, with *R* near 1. Bars show the average steady-state distribution of 1000 runs where *L*/*N* value is chosen in the [0.45, 0.55] interval and the error bars are the standard deviation. (**B**,**D**) Empirical data from ref.^3^ for ‘hard’ and ‘soft’ news respectively. (**E**) Other empirical examples from ref.^5^. (**F**,**G**) Theory and empirical data^3^ respectively, for the percentage of ties per individual type. (**H**,**I**) Theory and empirical data respectively for the time-dependent voting network of ref.^11^. (**J**,**K**) Theory and our empirical data (see Methods and Data) respectively for the relative fraction of the two extremes in the Colombian peace process as they evolve over time during 2016–2017.

This same challenge of understanding the *dynamics* of division and hence extremes (as in Fig. 1), and the impact of external information, is important in many disciplines–from physical and chemical systems^15^ to social, economic, ecological and political domains^4,16–31^. Even if a community appears relatively stable when observed from the outside, there can be a complex underlying ecology of beliefs and opinions bubbling just below the surface, driven by feedbacks based on past history and changes in relationships. Just as a liquid may then suddenly boil over without any apparent warning, extremes can appear from out of nowhere. Foundational works in economics by Arthur^21^, in physics by Halpin-Healy^22,23^, in ecology by Gavrilets^24^, in social science by Epstein, Axtell^25^ and Hedstrom^26^, in political science by Lazar^4,18,19^, and in psychology by Forsyth and Lewin^27,28^ and others^29–31^, suggest that addressing this challenge will therefore require the development of minimal, generative theories of the population’s out-of-equilibrium dynamics, even though any such theory will by necessity be a cartoon version of the real-world system^4,18,21–23,29–36^.

**Figure 2:**
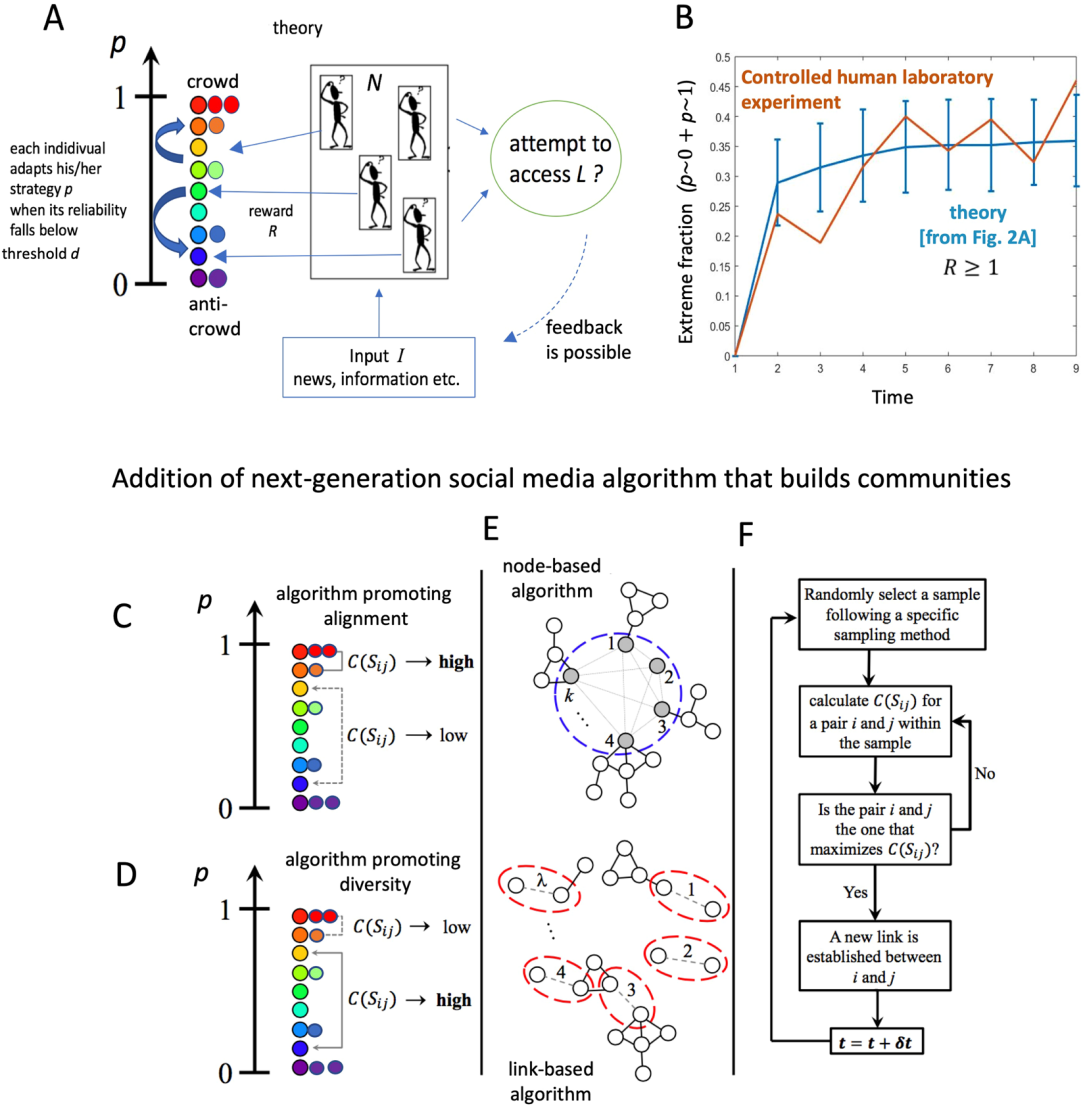
(**A**) Our dynamical theory describes an evolving population of boundedly-rational individuals^21^ pursuing individual gain in a setting where there are winners and losers. Each sporadically receives information *I* and decides how to act using their individual *p* value, and changes it when it becomes unreliable. The distribution of *p* values at time *t* is the heterogeneity distribution *P*(*p*, *t*). (**B**) Evolution of polarization in our controlled laboratory experiment (orange line, see Methods and Data) and our theory (blue line), with *R* ≥ 1. The theoretical curve is an average over 1000 runs where the *L*/*N* value is chosen in the [0.45, 0.55] interval, while the error bars represent the standard deviation. (**C**,**D**) Two next-generation social media algorithms which actively connect individuals with the aim of promoting alignment and diversity respectively. See Methods and Data for meaning of *C*(*S*_*ij*_). (**E**) Our results are insensitive to how these two algorithms are implemented, i.e. choosing *k* individuals (nodes) or *λ* links. (**F**) Example flow-chart of these two algorithms.

**Figure 3:**
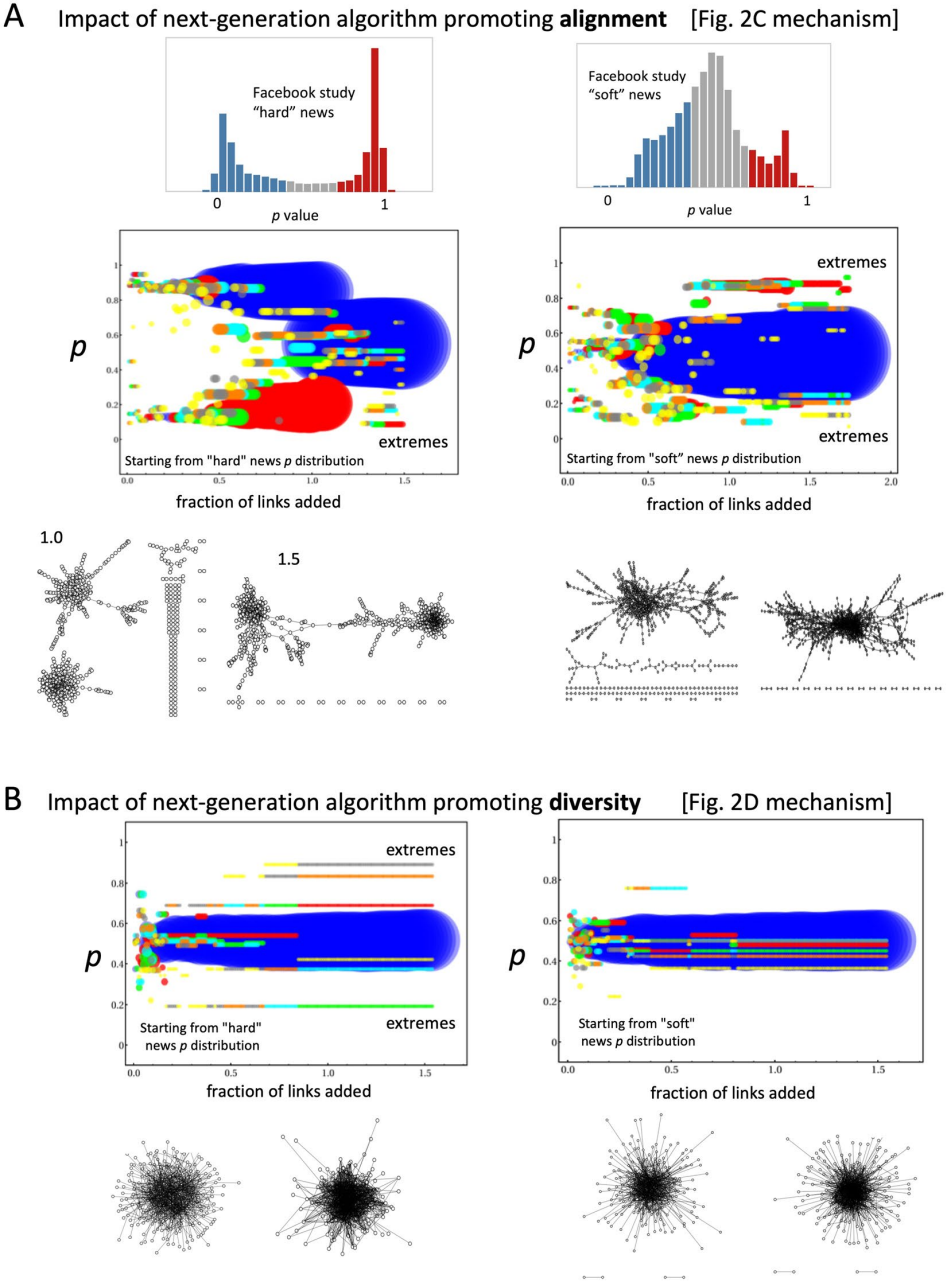
(**A**) Impact of adding links using next-generation algorithm promoting alignment (from Fig. 2C) on the heterogeneity distribution from Fig. 1B (left-hand side) and from Fig. 1D (right-hand side). Middle panels show circles whose center sits at the average *p* value for each of the seven largest clusters that are formed, and whose radius is proportional to the number of individuals in that cluster. Results shown as a function of the increasing fraction of links added. Different colors are used to denote different clusters. In both cases, small isolated clusters appear with more extreme average *p* values away from *p* = 0.5 even when there is a single large cluster formed around *p* = 0.5 and even though the fraction of links added is greater than 1.0 so that each individual is included in at least one link on average. Lower panels show snapshots of the actual network, at two values of the added-link fraction. The network for the ‘hard’ news heterogeneity distribution (left-hand side) shows that even the single large cluster that exists at added-link fraction 1.5 is actually internally highly segregated. It requires only one weak link to be broken in order to fragment the cluster into two separate pieces. (**B**) Same as A, but for the next-generation algorithm promoting diversity (from Fig. 2D). In this case, the main cluster forms quickly around *p* = 0.5, but it leaves a number of small isolated clusters with more extreme average *p* values that again survive until a surprisingly large fraction of links is added (≫1.0).

Here we present such a minimal yet generative dynamical theory (Fig. 2) which helps quantify when extremes emerge and how they evolve dynamically in time. It is supported by empirical data from controlled human laboratory experiments (Fig. 2B and Methods and Data) and a variety of real-world settings (Fig. 1). It reveals the underlying dynamical process as a many-body collective interaction between crowds and anti-crowds (Figs 1 and 2B). Each individual can adapt his/her belief and hence behavior (represented in a minimal way by a continuous *p* value where 0 ≤ *p* ≤ 1) in time according to its success, and hence the heterogeneity within the population (i.e. distribution *P*(*p*, *t*)) evolves over time. By contrast, most existing models of opinion formation maintain the heterogeneity distribution as fixed while allowing the location or connectivity to change in some spatial network, or they consider individuals having a discrete, binary state (e.g. 0 or 1, spin up or spin down) which can fluctuate while the network location or connections remain fixed^30,34–36^. Such models cannot reproduce or explain the *time-dependent* evolution and shapes of *P*(*p*, *t*). We then use our generative dynamical model to predict that the addition of next-generation social media enhancements (Fig. 2C,D) akin to those recently proposed by Facebook, will likely generate a new form of so-called explosive percolation^37,38^ within the population (Fig. 3). Although these algorithms can eventually connect together the majority of the population, we show that the process will likely generate *new* pockets of isolated extremes (Fig. 3).

## Results

### Our generative dynamical theory

Our dynamical theory (Fig. 2A) builds on the formal structure introduced by economist W.B. Arthur^21^ to explore how humans operate when there is no obvious deductive, rational solution for how they each should act^21^. We consider an arbitrary number *N* of heterogeneous, adaptive individuals who are going through life in pursuit of a piece of the common ‘pie’ of size *L*, i.e. each participant is effectively in pursuit of a common resource or benefit which is neither so small that nobody can access it, nor so large that there is no need to try. Most importantly, there are winners and losers just like in many aspects of everyday life. Indeed, all that is needed for the results shown in Fig. 1 to emerge from the model, is the perception that others may be winning more than you are and hence you are motivated to keep trying. It does not matter in the calculation what the abstract resource or benefit is exactly: it could be material in a socioeconomic context, or reputation, prestige or influence in some sociopolitical context, and the population could be a particular sector of society or an entity, or a geographic population such as a city, state or country etc. Interestingly, recent work by Wake *et al*.^39^ indicates the common population of neurons that processes social (e.g. good reputation) and non-social (e.g. monetary) rewards–a finding which goes beyond activation overlap and hints at a common neural mechanism in all humans. The Discussion (see Sec. III) discusses empirical support for our model from controlled laboratory experiments, from controlled real-world experiments, and from other uncontrolled real-world phenomena–as well as interpretation of the model in the various real-world settings of Fig. 1.

In the language of the model, each individual in the population sporadically receives an information update *I* via news or social media source, concerning economic, political and/or social prospects for the population. Despite the mature age of fragmented media^8^, the landmark study of King *et al*.^7^ shows that such common information (*I*) remains more influential, relevant and connected to a broad cross-section of the population than might otherwise have been thought. The collective dynamics in our model turn out to be insensitive to whether *I* is presented as good (which we encode as 1, suggesting that individuals should step up their efforts to chase *L*) or bad (which we encode as 0, suggesting that individuals should conserve their efforts until the next *I* appears) or if *I* is true or fake, or anything in between: it only matters that the information *I* is the same for all individuals. We also do not need to assume this is some zero-sum game or that *L* or *N* don’t change in time, but for simplicity we focus on *L*/*N* ~ 0.5 since this is the situation in everyday language of the glass being half-full or equivalently half-empty, meaning that there is no *a priori* bias in terms of how individuals should act. Hence individuals need to strategize as to when to step up their efforts (e.g. spend extra energy, money) in pursuit of *L*. Each individual uses his/her current strategy *p* to decide how to interpret *I* (i.e. good or bad, true or fake) and hence decides whether it is now worth stepping up to chase *L* and hence risk wasted efforts or not. Each strategy 0 ≤ *p* ≤ 1 is the probability that the individual takes *I* at face value and hence acts accordingly: if *I* = 1, then with probability *p* the individual subsequently chases *L* on the basis that prospects are good and so it is worth the risk. If *I* = 0, then with probability *p* the individual does not risk wasted efforts chasing *L*. During the time until the next *I* arrives and the process repeats, if the total number of individuals actively chasing *L* is less than *L*, those individuals consider themselves as having won and hence assign a reward *R* ~ 1 to the reliability score of their particular *p*, while those who chose not to chase *L* assign a unit penalty to the reliability score. If the total chasing *L* is more than *L*, those individuals assign a unit penalty to the reliability score, while those who chose not to chase *L* assign a reward *R* ~ 1 to the reliability score. We can equivalently consider individuals as gradually losing patience with their *p*, and hence the reliability score drifts steadily downwards. If an individual’s *p* has a reliability score that falls below some negative value *d*, he/she adapts by changing their *p* to a new value either side of the old one (without bias). This setup builds directly on prior work on evolutionary adaptive systems featuring self-segregation and clustering when individuals adapt their behavior according to past experiences^40–45^, and studies on random walks with time-dependent probabilities^46,47^. We have checked that our findings are insensitive to the precise choice of boundary condition, e.g. whether the new *p* value is chosen assuming reflective or periodic boundary conditions at *p* = 0 and *p* = 1, does not change the conclusions. The key ingredient is simply the ability of the *p* values to collectively evolve.

### Comparison with real-world data sets

Figure 1 shows the favorable comparison between our minimal dynamical theory and real-world data–not only for snapshots and time-averages where division develops (Fig. 1B,E,G) and where it does not (Fig. 1D), but also for the *dynamical* evolution prior to the emergence of extremes (Fig. 1I,K). Figure 2B shows similarly good agreement between this theory and the empirical dynamical evolution for the case of our controlled laboratory experiment on a smaller population (see Methods and Data). Numerical simulation of our dynamical theory (Fig. 2A) shows that the heterogeneity distribution *P*(*p*, *t*) evolves in time in a highly non-trivial way. To the extent that an individual’s *p* value captures his/her ideology, *P*(*p*, *t*) provides a prediction for how the population will collectively act at any given time *t* in terms of, for example, sharing of particular material online or voting. This is supported by the empirical agreement shown in Figs 1 and 2B. For example, our dynamical theory is able to reproduce the details of the observed ties from ref.^3^ (Fig. 1F,G) simply by tracking through time the people with whom each individual shares a similar *p* value.

Leaving to the Discussion (Sec. III) the interpretation of our model for the different scenarios in Fig. 1, we note that the model output captures the emergent dynamics of the polarization extremes exhibited by the U.S. congressional voting data (Fig. 1H,I), by the Colombian peace process data (Fig. 1J,K) and by our controlled laboratory experiments (Fig. 2B). In particular, it reproduces the novel plateau-like structure that emerges in Fig. 1K and is hinted at in our experiments in Fig. 2B. This dynamical bottleneck represents a temporary slowing-down of the polarization process, which in turn means that there is an extended period of time during which the polarization changes little, and hence during which interventions could be made to try to reverse the polarization process. This is the first report of such a societal polarization bottleneck that we are aware of. Whether the population *P*(*p*, *t*) develops a steady-state polarized U-shape as in Fig. 1A,B, or an inverted U-shape as in Fig. 1C,D, is dictated by the reward to penalty ratio *R*. A large enough *R* (i.e. *R* ≥ 1) prevents adaptation from being too fast and drives the formation of similarly-sized groups around the extremes *p* ~ 1 and *p* ~ 0 (crowd and anti-crowd). The closer individuals are to *p* ~ 1 and *p* ~ 0 respectively, the more likely they are to take opposite positions from each other. When *R* < 1^41–47^ an inverted U emerges (Fig. 1C,D). This provides a parsimonious explanation for the findings of ref.^3^ since ‘hard’ news (Fig. 1B) is directly relevant to the current socioeconomic climate and hence will be considered as relevant to an individual in deciding whether to pursue *L* at that moment, and hence *R* would be larger which is consistent with *R* ≥ 1 (Fig. 1A). ‘Soft’ news (Fig. 1D) is not directly relevant to the current climate and hence will play less of a role in whether an individual decides to pursue *L* at that moment: hence *R* would be smaller, consistent with *R* < 1 (Fig. 1C). We have checked that Kolmogorov-Smirnov goodness-of-fit tests generate goodness-of-fit P values that are consistently higher than 0.1 when comparing our model’s steady state distribution *P*(*p*) and that of Facebook study (Fig. 1B,D), for both cases (see Methods section).

### Our model’s robustness to parameter choices

In contrast to the key role played by *R*, the emergence of the polarized U-shape is insensitive to other parameters in the model, i.e. it is not sensitive to individuals’ threshold value *d*, or the size of the change of *p* upon adaptation, or the absolute values of *L* or *N* with *L*/*N* around 0.5, or the nature of the information *I*. It is also not sensitive to (1) whether the information *I* is fabricated to provide artificially high levels of ‘good’ news (e.g. all 1’s) or ‘bad’ news (e.g. all 0’s), or whether it is true or false, or whether it is released in a certain sequence; (2) whether we include population heterogeneity from the outset by randomly assigning *p* values, or set them all equal to 0.5 to mimic maximum initial uncertainty of all individuals; (3) the origin or precise nature of *I*; (4) the amount of clock-time that passes between the new arrival of information *I*; (5) the absolute value of the reward and penalty (only the ratio *R* is relevant); (6) the way in which individuals adapt and hence choose a new *p* value, as long as they do so independently; (7) whether different sectors of the society receive different *I* and hence exist in different news bubbles. As long as each bubble remains self-contained and has the same *R*, each U or inverted U will just add together and hence preserve the same shape; (8) other ‘bubble’ mechanisms, such as allowing individuals with similar *p* values to mimic each other. This applies even if the population gets broken up into only three bubbles. In short, while any of these factors may impact the way in which *P*(*p*, *t*) evolves and the time it takes to reach a steady-state, they do themselves dictate whether the final *P*(*p*, *t*) is U-shaped or not. Since a U and inverted U are symmetric around *p* = 0.5, even a switch in definition of what is extreme ‘left’ and what is extreme ‘right’ would not change the resulting *P*(*p*, *t*). We also note that generalizing the characterization of people’s strategies and hence ideologies from a single *p* value to a vector of attributes of arbitrary length, and/or allowing *I* to be a vector, does not change the emergence of crowds and anti-crowds at the extremes^45^.

### Next-generation social media algorithms

Given Facebook’s recent announcement of next-generation features that will actively create new bonds (links) between people in order to build new communities, we now build on the above framework to explore the likely impact that such social bonding algorithms (Fig. 2C,D) will have, assuming that they start from the present-day *P*(*p*, *t*) profiles shown in Fig. 1B,D^3^. Both the social bonding link formation and the *p* adaptation should operate simultaneously in the real world, and may even feed back on each other. However, unraveling such a complex coupled scenario will at the very least require understanding of the two mechanisms acting separately. Hence for simplicity, we here consider the limit where the social bonding link formation is fast compared to the *p* adaptation, i.e. *P*(*p*, *t*) ≡ *P*(*p*) and is static. Therefore the simulation starts from the present-day *P*(*p*, *t*) profiles shown in Fig. 1B,D^3^ and there is no further evolution of *P*(*p*, *t*) due to individuals winning and losing. We leave a study of both mechanisms acting simultaneously for future study. The alignment-algorithm (Fig. 2C) links together individuals based on the similarity of their *p*, and hence promotes the growth of clusters with high internal alignment. The diversity-algorithm (Fig. 2D) links together individuals based on the dissimilarity of their *p*, hence promotes the growth of clusters with high internal diversity. We have checked that our results are insensitive to exactly how these algorithms operate (Fig. 2E shows two obvious implementations in which either a candidate individual (i.e. node) or link is selected, while Fig. 2F shows the flow diagram).

Figure 3 shows that although both algorithms are designed with the intention of pulling together the population, several unexpected and potentially undesirable features emerge as the links are added. First, isolated residual clusters arise with an internal average *p* value well away from the population average of *p* = 0.5. Indeed, for the alignment-algorithm with either the ‘hard’ or ‘soft’ news *P*(*p*), and for the diversity-algorithm with the ‘hard’ news *P*(*p*), these isolated clusters are extreme in that their average internal *p* value is very close to the extremes *p* = 0 and 1. This is particularly surprising for the alignment-algorithm in the case of ‘soft’ news, since the ‘soft’ news *P*(*p*) population starts off with an average *p* ~ 0.5. Second, these extreme clusters persist even when the fraction of links is ≫1.0 and hence each individual has on average at least one new link. The fraction of added links is the ratio of the number of added links from the social bonding process in Fig. 2E compared to the total number of possible links, and it is the quantity plotted on the horizontal axes in Fig. 3. Third, even though links are added smoothly and monotonically by the algorithms, the cluster evolution shows an abrupt temporal evolution akin to explosive percolation^37^. Fourth, even though at high enough link fraction ≫1.0 the population is largely united into a single cluster, the network comprising the largest cluster for the ‘hard’ news diversity-algorithm is still internally highly segregated (see Fig. 3A left lower panel) meaning that it requires only one weak link to be broken in order for the population to fragment into two disconnected pieces.

### Mathematical analysis

We now provide mathematical analysis that explains and quantifies when and why subpopulations emerge at the extremes, and hence our findings in Fig. 1. We adopt three complementary perspectives which have been used successfully for physical systems: few-body, *N*-body statistical, and large-*N* dynamical. (1) Few-body: Since three is typically the minimum number of degrees of freedom for which complex dynamics can arise in a system, consider *N* = 3 individuals (or three groups sharing a common p value) and three possible *p* values: 0, 0.5 and 1. Halpin-Healy^22,23^ showed how such a ‘three-body’ theory in a comparable setting of conformity and dissent can provide a powerful quantitative description. Computer simulations of this *N* = 3 version of Fig. 2A reproduce similar U and inverted U-shapes as for general *N*. There are 3^3^ = 27 possible microstates (i.e. arrangements of the *N* = 3 individuals among the 3 *p* values)^41^, as shown in the Supplementary Information (SI). A microstate (*n*_0_, *n*_0.5_, *n*_1_) denotes *n*_0_ individuals with *p* = 0, *n*_0.5_ with *p* = 0.5 and *n*_1_ with *p* = 1, and each provides a certain average payoff per individual after accounting for rewards and penalties. For *R* = 1, the highest payoff microstates are (1, 1, 1) which is 6-fold degenerate, i.e. 6 arrangements of the individuals 1, 2 and 3, and (1, 0, 2) and (2, 0, 1) which are each 3-fold degenerate. Even though the population macrostate (U-shape as in Fig. 1A) appears static, the population continually transitions between these 12 microstates since individuals keep sporadically changing their *p* values–albeit at different rates–since there is no *p* value which is so good that it guarantees never needing adaptation. We can therefore take an average over time, to provide a theoretical prediction of the imbalance in heterogeneity. Assuming that all these microstates are visited equally, it follows that *P*(*p*) should have a U-shape in which *P*(0) = *P*(1) = 2.5*P*(0.5). Since the same is true for any set of three individuals, the general *N* population will also have a U-shape. Though the ratio is larger in the continuous-*p*, large-*N* case, this finding is consistent with the simulations (Fig. 1A). Moreover as in the simulations with large *N*, individuals with *p* ~ 0 and *p* ~ 1 retain their *p* value for a very long time while those with *p* ~ 0.5 continue to adapt most quickly. *R* > 1 behaves similarly. *R* < 1 favors microstates such as (0, 3, 0) and hence an inverted-U. (2) *N*-body statistical: Counting the probabilities for how adaptation takes individuals in and out of a specific region of *p*-space, an argument parallel to that of ref.^43^ (see SI) yields the following general expression:

1 $$P(p)=A{[\frac{1}{2}-p{F}_{N}^{ < }-\mathrm{(1}-p){F}_{N}^{ > }+2p\mathrm{(1}-p){G}_{N-1}]}^{-1}$$

where explicit expressions for ${F}_{N}^{ < }$, ${F}_{N}^{ > }$, *G*_*N*−1_ are derived in the SI, and *A* is determined by the normalization condition that *P*(*p*) integrates to unity. Despite their complex mathematical forms, the terms appearing in Eq. (1) have a simple interpretation that elucidates when and why subpopulations emerge at the extremes. Consider an individual who is merely observing this theoretical ‘rat race’ and hence not adding to the demand for *L*. The probability that their *p*-value wins at any given instance, is given by $\frac{1}{2}$ corrected by the second and third terms on the right-hand side. The fourth term is the one that accounts for their own impact in the system, just like a driver adds to the likelihood of a jam by being present on the road or a buyer inadvertently raises the asking price of an item simply by showing interest. When it is large enough, it can cause the inverted U-shape *P*(*p*) to flip into a U-shape *P*(*p*). (3) Large-*N* dynamical: Using an argument paralleling ref.^44^, it can be shown that *P*(*p*, *t*) in the large-*N* and continuous-time limit, satisfies a generalized diffusion equation $\frac{\partial P(p,t)}{\partial t}=\frac{{\partial }^{2}f[P(p,t)]}{\partial {p}^{2}}$ where *f*[..] denotes a functional of *P*(*p*, *t*) and hence describes the evolution of *P*(*p*, *t*) in time. Additional analysis then becomes possible using the time-dependent random-walk work of refs^45–47^. Though *P*(*p*, *t*) cannot be obtained in closed form, the competition between the emergence of a U-shape and inverted U-shape in the long-time limit is reflected in the fluctuations (variance) given by $N{\int }_{0}^{1}\,p\mathrm{(1}-p)P(p,t)dp$, which has extrema when *P*(*p*, *t*) is localized around *p* = 0.5 and when *P*(*p*, *t*) is localized around *p* = 0 and *p* = 1. The time-dependence of the polarization can be captured by a mean field-like equation for the rate-of-change of the fraction *x*(*t*) of the population in the crowd and anticrowd: *dx*/*dt* = *r* + *a*(*x* − *x*_0_)^2^ where *r* is the average growth rate, and the quadratic term is a self-interaction term. We estimate ${x}_{0} \sim \frac{1}{3}$ which is the approximate fraction of the population not in the crowd or anticrowd when *P*(*p*, *t*) is flat. For *r* > 0, there is a saddle-node ghost at $x \sim {x}_{0}$^48^ hence *x*(*t*) initially increases fast from $x\mathrm{(0)} \sim 0$, then forms a bottleneck, plateau shape around $x(t) \sim {x}_{0}$ before accelerating again–as observed in the real population polarization during the Colombian peace process in Fig. 1K, in the controlled human laboratory experiments in Fig. 2B, and in our dynamical theory in Figs 1J and 2B. The time spent by the population in the bottleneck can be obtained by integrating *dx*/*dt* to give approximately $a\pi /\sqrt{r}$ which diverges as *r* → 0. This suggests that even in a population that is in the process of polarizing, there will be an extended intermediate interval of time during which the system effectively sits as a tipping-point, during which minimal intervention could be used push the system polarization in the other direction and hence prevent the emergence of extremes.

## Discussion

While Schelling’s segregation model (SSM)^34–36^ also produces U-shapes, the underlying mechanisms and assumptions are completely different: in SSM, individuals locally try to be in the majority whereas in the present dynamical theory they are competing for *L* and do not require any local geographic connectivity; in the SSM the decision is made based on the present state of the system and is deterministic, while in the present dynamical theory the decision is stochastic based on a probability *p*; in the SSM, the individuals have no memory whereas in the present dynamical theory they record the scores of their *p* values, and also can include memory of the past without changing the conditions under which a U-shape emerges; the SSM is scale-dependent, i.e. the segregation emerges depending on how the agents choose their boundaries which implies local alignment rules, while the present dynamical theory is scale-free and independent of any geometry; the present dynamical theory can produce a U-shape or an inverted U by varying *R*, but in the SSM even if all individual agents have a strict preference for perfect integration, best-response dynamics may lead to segregation.

Of course, there are limitations in both our study and the justification of our modeling setup. This is the price we pay for a mathematically tractable model. However, though they are obviously an oversimplification, the mechanisms in Fig. 2C,D do at least provide plausible representations of next-generation algorithms, since they are a generic mechanism for connecting together individuals. Concerning Fig. 2A, there is–in addition to Arthur’s original real-world observation^21^ and the general weight of support for agent-based models^25,26^–the following empirical evidence that this provides a plausible representation of human behavior in everyday socioeconomic settings: (1) The controlled laboratory experiments carried out by one of us^40^ featuring the same setup of *N* individuals repeatedly deciding whether to access a common resource *L* < *N*. This controlled laboratory experiment differs from Axelrod’s Prisoner’s Dilemma tournaments^49^ in that it has a formal structure, it provides a tangible incentive for the individuals, and it uses the same subjects throughout. It makes no a priori assumptions about the structure of the learning dynamics. By focusing on the choice of a strategy as in our dynamical theory, it also differs from the standard economics experiment in which a subject chooses a single action during a round of play^50^. In accordance with our dynamical theory, these experiments show that individuals are heterogeneous in terms of strategies used; that they adopt different strategies over time based on their past success; that they show a tendency over time to move away from more complex mixed or conditional behaviors toward simpler strategies given *R* ≥ 1, i.e. ‘always’ in ref.^40^. which is akin to *p* ~ 1 and *p* ~ 0 and which also happen to become the most successful strategies in our theory (see later); that individuals do not manage to collectively find a more optimal solution (e.g. implicitly taking turns) but instead continually compete. A different set of experiments which also support these mechanisms, was carried out by Stanley *et al*.^10^. (2) The controlled real-world experiments of Konstantakopoulos *et al*.^51–53^ feature *N* individuals trying to access limited common lighting *L* < *N* and being rewarded accordingly. Though now in a real-world office setting, the findings are again consistent with our dynamical theory. (3) The uncontrolled real-world phenomenon of financial trading, in particular refs^54,55^, again confirms that, like our theory, the most popular and successful strategies turn out to be the ‘always’ type (e.g. momentum akin to *p* ~ 1 and contrarian akin to *p* ~ 0). Moreover, this was shown both for the case of humans choosing strategies when trading for themselves, and when choosing algorithms to write for machine trading. Other empirical studies with similar results include studies of drivers repeatedly deciding whether to access a potentially congested route^56^.

Despite the empirical support for our model coming from controlled laboratory experiments^40^, controlled real-world experiments^51–53^, and uncontrolled real-world phenomena^54,55^, the connection between the mechanisms of our model and the multiple real-world scenarios of Fig. 1 requires closer scrutiny. The very different real-world examples in Fig. 1 have in common that each broadly concerns a contentious political or societal setting that impacts individuals’ daily lives and for which there are two significant sides that can develop. Our argument in favor of the plausibility of our model is that for each of these cases, each individual at any one time has a particular opinion or position (i.e. *p* value) in mind, and goes through their daily life making decisions based loosely on it. This is the socioeconomic or sociopolitical scenario discussed and justified by psychologists and economists including Arthur and others^21^ as being a common feature of everyday life, where not everyone at any one moment can feel that things are going well for them–or equivalently that others are doing better than them. While the precise reason that any given individual may feel a winner at any given moment may be exogenous, often it is not since many everyday scenarios (i.e. promotion, pay rise, uncongested commute) necessarily benefit some and not others, hence there are some individuals who will at that moment perceive themselves as winners and some who will not. It is common to hear people then blame the incumbent ‘system’ and/or government when things are not going well for them or when they feel that others are winning more than them, even if it is just their daily commute: and this is all that matters for our model to make sense in terms of them evaluating their opinion or position (i.e. *p* value). Hence the precise nature of the real-world decisions is not important, it just matters that in the real world not everyone feels a winner all the time. While this seems reasonable to the authors of this article, its validity is ultimately an empirical question that needs to be tested more thoroughly in the future as appropriate real-world data arises. However, it seems encouraging that the model reproduces the features of so many different examples in Fig. 1, and that these outputs come from a minimal model that, in essence, just relies on the simple feature of some people feeling like winners at any particular moment, and some who don’t. Most importantly, the feature in the model of a limited resource *L* does not need to be taken literally, and instead just provides a fictitious mechanism for reproducing the collective winner-loser dynamics. Also there do not need to be more losers than winners (i.e. it is not simply a contrarian game) since *L* can be larger than *N*/2, as shown in Fig. S1, without changing the emergence of extremes near *p* = 0, 1. Similarly, the *p* of each individual can be interpreted very broadly as their ‘way of thinking’ for deciding how to act in everyday challenges such as competing in a work context, or with neighbors, or simply trying to have an easy commute. When things go wrong for them individually, they tend to blame the system and/or incumbent government and penalize *p*, and when things go right they reward their *p*.

Given this, we feel that it is therefore reasonable to compare the model’s *P*(*p*, *t*) with the output of the Facebook study (Fig. 1B,D) showing the polarization of individuals’ positions. The model should also therefore crudely mimic the voting scenario in Fig. 1E if we assume that the more certain an individual is about leaning to the right or left (*p* → 1 or *p* → 0) then the more likely they are to make the effort to go and vote. Similarly, the model’s *P*(*p*, *t*) should crudely mimic the political contribution scenario in Fig. 1E if we assume that the more certain an individual is about leaning to the right or left (*p* → 1 or *p* → 0) then the more likely they are to contribute their own money. Likewise for the congressional voting in Fig. 1H, each congressional voter can at each moment feel internally that, thanks to their position *p* in front of the available information *I*, things are going well or badly for them and hence they might, after an extended bad period, change their *p* value. For the peace process in Colombia in Fig. 1K, which initially seems to have the most remote connection to the model, it is also reasonable that each citizen has a position *p* with regards the peace process at any one time. This position *p* would then reasonably impact the type of decisions and behavior that they exhibit at that time–for example, someone supporting the government’s peace plan (*p* ~ 1) might invest in land in a current battleground with the belief that it would soon increase in value, or arrange to visit that place with their family, or simply venture out to a restaurant in a previously dangerous area. These were indeed rather common stories in Colombia given the large amount of Colombian territory that was under guerrilla control during the war, and the limitations on daily life that it created. If a large crowd of people then acted similarly, and hence showed significant optimism in apparent support of the government’s peace plan, the guerrilla (FARC) would then sometimes retaliate by carrying out a destabilizing violent attack in that area. The crowd of optimists aligned with the government’s peace plan (*p* ~ 1) would in that case feel like they lost while the anti-crowd against the government’s peace plan (*p* ~ 0) would feel vindicated, i.e. they would feel that their thinking was correct and hence reward their *p*. Faced with the news *I* of events and attacks each day, each individual then each day rewards or penalizes their way of thinking about the peace process (i.e. their *p*), hence making the peace process in Fig. 1K very crudely akin to the model’s mechanism.

We fully acknowledge that this correspondence between the model and specific real-world scenarios requires more study, and believe that it is ultimately an empirical question. However in this regard, we note that in addition to the encouragingly similar shapes appearing for the real data and the model, the model also passes the more stringent analysis in Fig. 1F,G derived from the network dynamics of the individuals. Future analyses against other empirical data can help strengthen confidence in our model.

In the future, as new features are proposed to enhance the technology and connectivity of online media, more specific types of network connections can be added into our model simulations and incorporated into the above mathematical analysis within a mean-field approximation. This will allow predictions to be made about the likely impact of new social media features on the population polarization and the emergence of extremes, and hence appropriate adjustments could in principle be made before it is launched.

## Methods and Data

Our controlled laboratory experiment (Fig. 2B) to test our model theory (Fig. 2A) was implemented by one of us with full detailed documentation available online (see ref.^40^ for full details). The computer simulation of our dynamical theory in Fig. 2A is straightforward and builds on existing software that is freely available for Arthur’s El Farol problem. To run simulations within any browser and without requiring any programming skills, see http://ccl.northwestern.edu/netlogo/models/ElFarol. For instructions on how to modify this simulation code, see http://citeseerx.ist.psu.edu/viewdoc/download?doi=10.1.1.60.7968&rep=&rep1&type=pdf. To calculate the average of the statistical P value between the Facebook study data (Fig. 1B,D) and our model, we selected random representative samples from the respective distributions and compute the goodness-of-fit (KS test) using the standard statistical package available in Mathematica 11. For each case (‘Soft’ and ‘Hard’ news), we tested 1000 different random samples of 100 entries each using different model realizations. For both cases, the average P value obtained was around 0.17. Our data from the Colombian peace process was collected by us, and comes from the public posts of the 218 Facebook collective profiles (i.e. groups and pages, but not private individual accounts) identified through online trawling, whose discussions were centered on the peace talks between the State and Marxist guerrillas. The data capture methodology copies exactly the approach introduced and discussed in depth in ref.^9^ of the main paper. The data was captured through Facebook’s API. Each collective was coded according to its polarity (i.e. supporters, opponents, or neutral discussants of the peace policies). The logs comprise more than 4 million messages, that were processed to identify the number of unique commenters (i.e. those who posted at least once), per month, per polarity. The next-generation algorithms in Fig. 2C,D are implemented as follows: The mechanisms of link addition follow directly from the relationship among the *p* values associated to the individuals (i.e. nodes) to be linked. This is quantified by the similarity *S*_*ij*_ between individual i and individual j which is defined as *S*_*ij*_ = 1 − |*p*_*i*_ − *p*_*j*_|. Thus highly similar individuals are close to each other in the p distribution, and otherwise for highly dissimilar individuals. The mechanisms of link addition depend on the coalescence function *C*(*S*_*ij*_). The alignment-algorithm (Fig. 2C) favors connecting similar individuals. The diversity-algorithm (Fig. 2D) favors connecting dissimilar individuals. A system following the alignment-algorithm tends to form clusters of alike individuals while diversity-algorithm tends to form clusters with unlike or complementary individuals. Hence, for the alignment-algorithm a coalescence function is defined as *C*(*S*_*ij*_) = *S*_*ij*_ while for the diversity-algorithm, *C*(*S*_*ij*_) = 1 − *S*_*ij*_. The present state, i.e. prior to any next-generation algorithms, has no additional links. At each timestep, a sample from the system is randomly selected and a new link is established between the pair of individuals that maximizes the coalescence function *C*(*S*_*ij*_). Though the sampling can be either of individuals (i.e. nodes) or links, the evolution of the network presents similar properties.

## Supplementary information

Supplementary Info

## Data Availability

All the data and described in this paper are freely available in either published papers as referenced, or on the Internet including via free online access to published PhD theses (specifically, those of co-authors Leady, Velasquez).
