# Supplementary material for: Emergent dynamics of extremes in a population driven by common information sources and new social media algorithms
Source: Sci Rep. 2019 Aug 15;9:11895. doi: 10.1038/s41598-019-48412-w (PMC6695450; doi:10.1038/s41598-019-48412-w)
Supplement: Supplementary file 1 — Supplementary Info [file 41598_2019_48412_MOESM1_ESM.pdf]

SUPPLEMENTARY INFORMATION (SI)
Emergent dynamics of extremes in a population driven by common information sources and new social media algorithms

N.F. Johnson[1,*], P. Manrique[2], M. Zheng[2], Z. Cao[2], J. Botero[2], S. Huang[2], N. Aden[2], C. Song[2], J. Leady[3], N. Velasquez[4], and E.M. Restrepo[4]

[1]*Physics Department, George Washington University, Washington D.C. 20052, USA*
[2]*Physics Department, University of Miami, FL 33126, USA*
[3]*Mendoza College of Business, University of Notre Dame, IN 46556, USA*
[4]*Elliott School of International Affairs, George Washington University, Washington D.C. 20052, USA and*
[*]*corresponding author: neiljohnson@gwu.edu*

These sections give additional details and make the paper and SI self-contained. Section 1 draws heavily from Ref. 39 of the main paper, while Section 2 draws heavily from Ref. 41 of the main paper. We are extremely grateful to T. Lo and P.M. Hui for their collaboration with the earlier development of this material.

1. MODEL IN FIG. 2A AND ANALYTICAL RESULTS FOR $N = 3$

The theory in Fig. 2A considers N individuals repeatedly using their individual strategy value p to choose between option 0 (don't chase L) or option 1 (chase L) as in the main text. We focus on two quantities, $P(p,t) \equiv P(p)$ and the lifetime of a particular p-value $T(p,t) \equiv T(p)$ in the asymptotic limit of large t. $P(p)$ is the distribution of p values, and $T(p)$ is the lifespan defined as the average length of time a p value survives between modifications. As discussed in the text, $P(p)$ and $T(p)$ are surprisingly insensitive to the nature of the information I. For example, I could be a record of past outcomes of any length. It just matters that it is a common information source. The simplest example of our system contains $N = 3$ individuals i, j, k and three discrete p values $p = 0, \frac{1}{2}, 1$, and hence $L = N/2$ means that L is large enough for one individual but not for two. There are $3^3 = 27$ possible configurations (p_i, p_j, p_k). For a given (p_i, p_j, p_k), the 8 possible decisions yield the expected gain. For example for $(p_i, p_j, p_k) = (0, 0, \frac{1}{2})$, i and j both choose 1 while k chooses 0 with probability $\frac{1}{2}$. Hence k wins with probability $\frac{1}{2}$ whereas i and j both lose. The net number of points gained per individual per turn, given by the points awarded minus the points deducted, is -1 for i, -1 for j, and 0 for k. The total is hence -2. Given that the maximum is -1 (there is a maximum of one winner) we see that $(0, 0, \frac{1}{2})$ is not optimal. Table 1 shows the various configuration types, or classes. The last column shows the average points per individual: $[-\frac{1}{2}]$ for class i) implies the average individual loses $-\frac{1}{2}$ point per turn, and would hence modify its p value (i.e. strategy) after time $2d$. Such strategy modification allows the system to sample the 27 configurations. Classes vi), vii) and viii) are optimal, having maximum points. To obtain the average distribution $P(p)$ and $T(p)$, we must average over all 27 configurations. Since some classes are more favorable (i.e., more points) we should weight the distributions in an appropriate way. In the extreme case of large weighting, we include only the optimal classes vi), vii) and viii), yielding $P(0) : P(\frac{1}{2}) : P(1) = 2.5 : 1 : 2.5$ and $T(0) : T(\frac{1}{2}) : T(1) = 5 : 1 : 5$. For zero weighting, we instead consider the system as visiting all configurations with equal probability regardless of points gained per individual; such a zero-weight averaging is similar to that for the microstates in a gas within the microcanonical ensemble and yields $P(0) : P(\frac{1}{2}) : P(1) = 1 : 1 : 1$ and $T(0) : T(\frac{1}{2}) : T(1) = 1 : 1 : 1$. For an intermediate case, whereby all classes are weighted by the average points per individual, we obtain $P(0) : P(\frac{1}{2}) : P(1) = 1.1 : 1 : 1.1$ and $T(0) : T(\frac{1}{2}) : T(1) = 1.5 : 1 : 1.5$. In fact, any sensible weighting which favors the more profitable configurations yields a non-uniform $P(p)$ and $T(p)$ as observed numerically. This implies that the population, by self-segregating, has also managed to self-organize itself around the most profitable configurations. We emphasize that the system is dynamic since the membership of the various configurations is constantly changing (i, j and k inter-diffuse) but $P(p)$ remains essentially constant. For general N, we can loosely think of i, j, k as three equal-size groups of like-minded individuals.

2. DERIVATION OF EQ. 1 AND EXPLICIT EXPRESSIONS FOR THE TERMS

Consider a certain moment in this steady-state regime. Let the information I say 1. The following arguments also hold if I says 0. We define $F_N(n)$ as the probability of there being n individuals choosing the same as I suggests. It follows from the central limit theorem that $F_N(n)$ will be an approximately Gaussian distribution with a mean $N\overline{p}$ and variance $N \int_0^1 P(p)p(1-p)dp$. Here $\overline{p}$ is the mean of p given by $\overline{p} = \int_0^1 pP(p)dp$, which is known if the distribution $P(p)$ is known. However, $P(p)$ is the unknown for which we are going to solve. In the steady state, $F_N(n)$ becomes identical to the probability of choosing either outcome since the two possible outcomes occur equally often on average when L is $N/2$. Following the approach of self-consistent mean-field theories, we consider the interaction between a particular individual and the rest of the population, presenting the formulation in a general way so that it can be readily generalized to different variations of our model. Specifically, consider the action of the k-th individual in the background of the other $N-1$ individuals. Let $G^k_{N-1}(n)$ be the probability of n individuals following I, given that there are only $(N-1)$ individuals participating (i.e. excluding the k-th individual). Then $F_N(n)$ can be written in terms of G^k_{N-1} as

$$F_N(n) = p_k G^k_{N-1}(n-1) + (1-p_k) G^k_{N-1}(n), \tag{1}$$

where $n \neq 0, N$. Here p_k is the p-value of the k-th individual at that moment. A value of n choosing "1" is achieved if the value of the $(N-1)$ individual background is $n-1$ *and* the k-th individual decides "1": this leads to the first term in Eq. (1). Alternatively the $(N-1)$ individual background is n and the k-th individual decides against "1", leading to the second term in Eq.(1). Let $\tau(p_k)$ be the winning probability of the k-th individual. Given the probability

$G^k_{N-1}(n)$, we can write

$$\tau(p_k) = p_k \sum_{n=0}^{(N-3)/2} G^k_{N-1}(n) + (1-p_k) \sum_{n=(N+1)/2}^{N-1} G^k_{N-1}(n). \tag{2}$$

Equation (2) says that the k-th individual wins if (i) the number choosing "1" is $(N-3)/2$ before he/she makes their move and he/she decides "1", thereby giving the first term or (ii) the number choosing "1" is above $(N+1)/2$ and he/she decides not to choose "1", thereby giving the second term. Since the k-th individual is only characterized by his/her p-value p_k, $\tau(p_k)$ can also be interpreted as the success rate of an individual using value p_k. It follows from Eq.(1) that

$$\begin{aligned}\sum_{n=1}^{(N-3)/2} F_N(n) &= \sum_{n=1}^{(N-3)/2} \left[p_k(G^k_{N-1}(n-1) - G^k_{N-1}(n)) + G^k_{N-1}(n)\right] \\ &= \sum_{n=1}^{(N-3)/2} G^k_{N-1}(n) + p_k G^k_{N-1}(0) - p_k G^k_{N-1}(\frac{N-3}{2}).\end{aligned}$$

Since $F_N(0) = (1-p_k)G^k_{N-1}(0)$, which follows from the consideration that nobody chooses "1" if the other $N-1$ individuals do not choose "1" and the k-th individual does not choose "1", we have

$$\sum_{n=0}^{(N-3)/2} G^k_{N-1}(n) = \sum_{n=0}^{(N-3)/2} F_N(n) + p_k G^k_{N-1}(\frac{N-3}{2}). \tag{3}$$

Similarly, we have from Eq.(1)

$$\begin{aligned}\sum_{n=(N+1)/2}^{N-1} F_N(n) &= \sum_{n=(N+1)/2}^{N-1} \left[p_k(G^k_{N-1}(n-1) - G^k_{N-1}(n)) + G^k_{N-1}(n)\right] \\ &= \sum_{n=(N+1)/2}^{N-1} G^k_{N-1}(n) + p_k G^k_{N-1}(\frac{N-1}{2}) - p_k G^k_{N-1}(N-1).\end{aligned}$$

Since $F_N(N) = p_k G^k_{N-1}(N-1)$, which follows from the consideration that all the individuals choose "1" only if all the other $N-1$ individuals choose "1" and the k-th individual chooses "1", we have

$$\sum_{n=(N+1)/2}^{N-1} G^k_{N-1}(n) = \sum_{n=(N+1)/2}^{N} F_N(n) - p_k G^k_{N-1}(\frac{N-1}{2}). \tag{4}$$

Substituting Eqs.(3) and (4) into Eq.(2), we obtain

$$\begin{aligned}\tau(p_k) &= p_k \sum_{n=0}^{(N-3)/2} F_N(n) + p_k^2 G^k_{N-1}(\frac{N-3}{2}) \\ &\quad +(1-p_k) \sum_{n=(N+1)/2}^{N} F_N(n) - (1-p_k)p_k G^k_{N-1}(\frac{N-1}{2}).\end{aligned}$$

Using Eq.(1) to express $G^k_{N-1}(\frac{N-3}{2})$ in terms of $G^k_{N-1}(\frac{N-1}{2})$ and $F_N(\frac{N-1}{2})$, we then obtain

$$\begin{aligned}\tau(p_k) &= p_k \sum_{n=0}^{(N-3)/2} F_N(n) + (1-p_k) \sum_{n=(N+1)/2}^{N} F_N(n) \\ &\quad +p_k \left(F_N(\frac{N-1}{2}) - 2(1-p)G^k_{N-1}(\frac{N-1}{2})\right) \\ &= p_k \sum_{n=0}^{(N-1)/2} F_N(n) + (1-p_k) \sum_{n=(N+1)/2}^{N} F_N(n) - 2p_k(1-p_k)G^k_{N-1}(\frac{N-1}{2}).\end{aligned} \tag{5}$$

Equation (5) separates $\tau(p_k)$ into 3 terms, which are interpreted as follows. Consider an "outsider", i.e. someone whose action does not affect the outcome but instead is only betting on which side is the winning room according to the probability p_k. His/her winning probability is given by the first two terms in Eq.(5). The third term gives the difference in the winning probability between an outsider and an individual who actually participates. This term is negative, reflecting the fact that an individual has a smaller probability of winning when he is actually participating in the game. Consider the case in which the background population is split evenly between "0" and "1": the k-th individual loses no matter what action he/she takes. Thus the third term represents this self-interaction term, or so-called market impact. The $p_k(1-p_k)$ factor means that the winning probability increases as p_k deviates more from the value 1/2, and it produces a symmetry about $p = 1/2$ in $T(p)$ and $P(p)$. Note that Eq.(5) also applies to the case when I says 0: hence it is independent of the dynamics of I. This further implies that the resulting $P(p)$ and $T(p)$ is insensitive to most of the parameters of the model, as claimed in the main paper. For L near $N/2$, 1 and 0 occur a similar number of times on the average. In this case, the summations in the first and second terms of Eq.(5) in the steady state yield the value 1/2 and hence $\tau(p)$ becomes

$$\tau(p_k) = \frac{1}{2} - 2p_k(1-p_k)G^k_{N-1}(\frac{N-1}{2}). \tag{6}$$

In order to express the right hand side of Eq.(5) entirely in terms of the function F, we use Eq.(1) to find $G^k_{N-1}(\frac{N-1}{2})$. From Eq.(1), we have

$$p_k G^k_{N-1}(n-2) + (1-p_k)G^k_{N-1}(n-1) = F_N(n-1). \tag{7}$$

Subtracting the equations obtained by multiplying Eq.(1) by $(1-p_k)$ and multiplying Eq.(7) by p_k, we can eliminate $G^k_{N-1}(n-1)$ to obtain

$$(1-p_k)F_N(n) - p_k F_N(n-1) = (1-p_k)^2 G^k_{N-1}(n) - p_k^2 G^k_{N-1}(n-2).$$

Repeatedly applying Eq.(1), we can eliminate $G^k_{N-1}(n-2)$, $G^k_{N-1}(n-3)$,$\cdots$ to obtain

$$\sum_{j=0}^{n}(-1)^{n-j}F_N(j)(\frac{p_k}{1-p_k})^{n-j} = (1-p_k)G^k_{N-1}(n)\ . \tag{8}$$

Similarly, if we apply Eq.(1) with increasing values of n instead of decreasing values of n, we obtain

$$\sum_{j=n+1}^{N}(-1)^{j-n-1}F_N(j)(\frac{1-p_k}{p_k})^{j-n-1} = p_k G^k_{N-1}(n)\ . \tag{9}$$

Although the results are exact, in practice it makes sense to use Eq.(8) for small p_k and Eq.(9) for $p_k \sim 1$. Using Eq.(8) or Eq.(9) for $n = \frac{N-1}{2}$ and substituting the result into Eq.(5), we obtain $\tau(p_k)$ entirely in terms of $F_N(n)$, and the label k becomes irrelevant. As mentioned, $\tau(p_k)$ can be regarded as the winning probability of an individual who is using strategy value p, and henceforth we denote it by $\tau(p)$ for simplicity. In order to obtain $P(p)$ from $\tau(p)$, we note that these two quantities are related. It turns out that the stationary distributions $P(p)$ and $T(p)$ are proportional to each other:

$$\frac{P(p)}{T(p)} = constant, \tag{10}$$

where the right hand side is a constant independent of p. Equation (10) follows from the balance between the fluxes of individuals into and out of a region in p-space in the steady state. Since an individual using a value p loses $(1-2\tau(p))$ points each turn, the lifespan $T(p)$ is given by

$$\tau(p) = \frac{|d|}{1-2\tau(p)}.$$

From Eq.(10), we have

$$P(p) \propto \frac{1}{1/2 - \tau(p)}, \tag{11}$$

with the proportionality constant determined by the normalization of $P(p)$ to $\int_0^1 P(p)dp = 1$. This means that

$$P(p) \propto [1/2 - p_k \sum_{n=0}^{(N-1)/2} F_N(n) - (1-p_k) \sum_{n=(N+1)/2}^{N} F_N(n) + 2p_k(1-p_k)G^k_{N-1}(\frac{N-1}{2})]^{-1} \tag{12}$$

which is exactly the form of Eq. (1) of the main paper, where

$$F_N^< \equiv \sum_{n=0}^{(N-1)/2} F_N(n) \tag{13}$$

and

$$F_N^> \equiv \sum_{n=(N+1)/2}^{N} F_N(n) \ . \tag{14}$$

and $G_{N-1} \equiv G^k_{N-1}(\frac{N-1}{2})$ is defined above. In the limit that L is very near $N/2$, and hence 1 and 0 occur a similar number of times on the average, the summations in the second and third terms in Eq. 12 in the steady state yield the value 1/2 and hence

$$P(p) \propto [2p_k(1-p_k)G^k_{N-1}(\frac{N-1}{2})]^{-1} \tag{15}$$

which is even simpler than Eq. 1 in the main paper.

It is hence straightforward to construct a general iterative calculation scheme for $P(p)$. The steps are the following: (a) assume a form for $P(p)$, (b) obtain $F_N(n)$ by evaluating $\overline{p}$ and the standard deviation from the assumed $P(p)$, (c) use Eq.(5) together with Eqs.(8) and (9) to obtain $\tau(p)$, (d) calculate $P(p)$ from $\tau(p)$ using Eq.(11) and the normalization condition, (e) check for convergence of $P(p)$ and, if necessary, repeat the steps until convergence is obtained. Note that Eq. (5) is employed since it is valid for all forms of initial guess for $P(p)$, including those which are non-symmetrical about $p = 1/2$. $P(p)$, when properly normalized, is not sensitive to N, while $T(p)$ depends on N. Results from our theory are in good agreement with numerical data. A further test of the theory is obtained by comparing results for $\tau(p)$ as a function of p with numerical data for different N. $\tau(p)$ provides a better test than $P(p)$ for the validity of any theory, since many forms of $\tau(p)$ can give rise to similar forms for $P(p)$. Simulations suggest that the correct $\tau(p)$ in the steady state, which follows from Eq.(5) (see also Eq.(6)), has the form $\tau(p) \sim 1/2 - \mathcal{A}(N)p(1-p)$ where $\mathcal{A}(N)$ is an N-dependent constant which decreases with N as $1/\sqrt{N}$. Such a scaling with N makes sense from random walk arguments.

3. COMPLEMENTARY RESULTS FROM OUR GENERATIVE DYNAMICAL THEORY

Figure S1 illustrates the ratio of extremes to neutrals as a function of the ratio of reward to penalty R and the resource level L/N. Specifically, Fig. S1 shows the ratio of the steady state distribution $P(p)$ for $p > 0.9$ and $p < 0.1$ to the steady state distribution $P(p)$ for $0.4 < p < 0.6$. Hence values of this ratio that are greater than 1, signal strong polarization. This situation generally arises for $R \geq 1$ where we find consistently high ratios (and hence strong polarization) for a wide range of L/N values. Even for $R < 1$, the polarization is significant: this can be understood by considering the null hypothesis that given L/N is near 0.5 individuals should throw a coin, hence generating a Gaussian-like $P(p)$ peaked around $p = 0.5$ and hence having an extremes-to-neutrals ratio near zero. As can be seen, this does not happen. In fact, the ratio is typically much larger, and so polarization exists. In short, our main conclusions are general.

4. ADDITIONAL DETAILS BEHIND THE NEXT-GENERATION ALGORITHM IMPLEMENTATIONS

We consider a population of N individuals as in Figs. 1B,1D which are yet to have any formal links introduced between them due to the introduction of next-generation algorithms. Now we imagine turning on the next-generation algorithms. Individuals (i.e. henceforth referred to as nodes) interact via character comparison which we call similarity (S_{ij}) which is defined as $S_{ij} = 1 - |p_i - p_j|$. A link is established between a node i and a node j if the coalescence

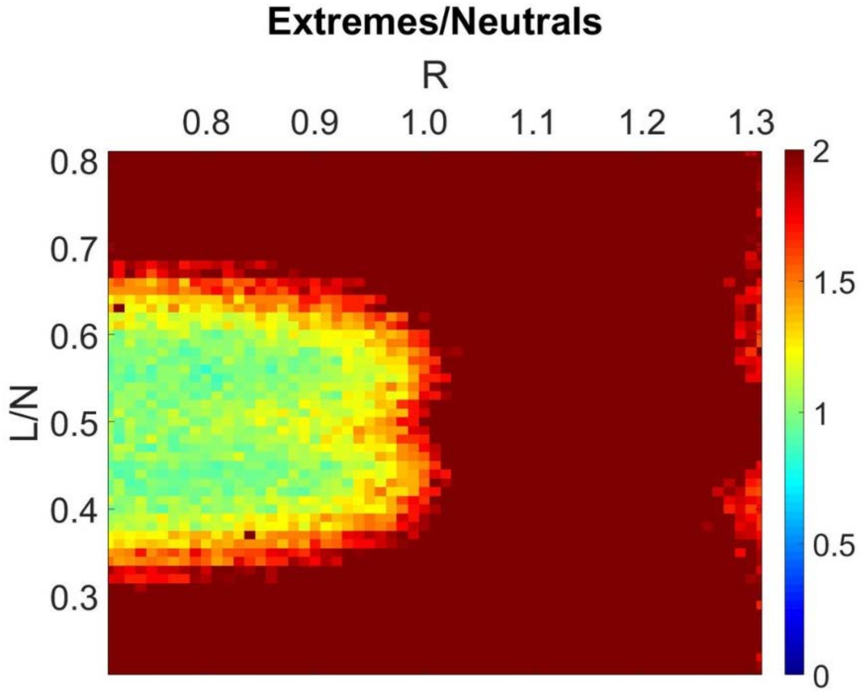

FIG. S1. Steady state ratio of extreme-to-neutral individuals as a function of reward to penalty ratio R and resource level L/N. The extreme-to-neutral ratio is computed from the steady state distribution $P(p)$ by taking $p > 0.9$ and $p < 0.1$ for extremes, and $0.4 < p < 0.6$ for neutrals.

function $\mathcal{C}(S_{ij})$ is maximum. This function inputs the values p_i and p_j associated to the nodes i and j, respectively. In the main paper, we look at two contrasting mechanisms for establishing a connection: one that benefits alike p connections (alignment) and the other benefits unlike p connections (diversity). We define a coalescence function for each mechanism as:

$$\mathcal{C}(S_{ij}) = S_{ij}, \quad \text{for alignment algorithm} \tag{16}$$

$$\mathcal{C}(S_{ij}) = 1 - S_{ij}, \quad \text{for diversity algorithm} \tag{17}$$

As in the approaches adopted in the network community (see Ref. 37 of the main paper) the connecting process starts by taking a series of random samples of specific sizes. Within each sample the connection that maximizes $\mathcal{C}(S_{ij})$ is established. We look at two different methods of sampling: *nodes* and *links*. The former selects a specific number of nodes while the latter selects links.

Nodes' (i.e. individuals') sampling method in Fig. 2E

We consider a sample of random unconnected nodes to be selected at every iteration. We call k the size of the sample and hence $k = 2$ constitutes the traditional random percolation process where two nodes are randomly connected at every iteration. This connects the suggested next-generation algorithm to work in the network community (see Ref. 37 of the main paper) in the sense that this next-generation algorithm generalizes the approaches of Ref. 37 to now account for individual heterogeneity. For $k \geq 3$, a character-based competition takes place. The pair of nodes within the sample of size k that maximizes $\mathcal{C}(S_{ij})$ is selected to establish a connection. The selected nodes can be either isolated or belong to different clusters. Thus, the system can change in every iteration until all the nodes are connected to each other in a single large cluster.

TABLE S1. Configuration classes showing the distribution of the three individuals (each denoted by x) and the average points awarded per time-step for each strategy-value p. Also given are the number of distinct configurations per class, and the average number of points per individual per time-step.

Class	$p = 0$	$p = 1/2$	$p = 1$	No. configs.	Ave pts/individual
i)	_	xxx[-1/2][-1/2][-1/2]	_	1	[-1/2]
ii)	x[-1/2]	xx[-1/2][-1/2]	_	3	[-1/2]
iii)	xx[-1][-1]	x[0]	_	3	[-2/3]
iv)	xxx[-1][-1][-1]	_	_	1	[-1]
v)	_	_	xxx[-1][-1][-1]	1	[-1]
vi)	x[1]	_	xx[-1][-1]	3	[-1/3]
vii)	xx[-1][-1]	_	x[1]	3	[-1/3]
viii)	x[0]	x[-1]	x[0]	6	[-1/3]
ix)	_	xx[-1/2][-1/2]	x[-1/2]	3	[-1/2]
x)	_	x[0]	xx[-1][-1]	3	[-2/3]

Links' sampling method in Fig. 2E

This method considers randomly sampling a specific number of potential links λ to compete for addition. For each potential link the function $\mathcal{C}(S_{ij})$ associated to its end nodes i and j is evaluated. Note again that the case of $\lambda = 1$ corresponds to the traditional random percolation process and for $\lambda \geq 2$ a character-driven competition takes place among the potential λ links. All of the potential links selected are nonexistent so in every iteration the system gains a new link.
